# Sensitivity analysis of biological Boolean networks using information fusion based on nonadditive set functions

**DOI:** 10.1186/s12918-014-0092-4

**Published:** 2014-09-05

**Authors:** Naomi Kochi, Tomáš Helikar, Laura Allen, Jim A Rogers, Zhenyuan Wang, Mihaela T Matache

**Affiliations:** 1 Department of Genetics, Cell Biology, and Anatomy, University of Nebraska Medical Center, Omaha NE 68198, USA; 2 Department of Mathematics, University of Nebraska at Omaha, Omaha NE 68182, USA; 3 Department of Biochemistry, University of Nebraska-Lincoln, Lincoln NE 68588, USA

**Keywords:** Information fusion, Node attributes, Signal transduction, Nonadditive set functions, Choquet integral, Sensitivity

## Abstract

**Background:**

An algebraic method for information fusion based on nonadditive set functions is used to assess the joint contribution of Boolean network attributes to the sensitivity of the network to individual node mutations. The node attributes or characteristics under consideration are: in-degree, out-degree, minimum and average path lengths, bias, average sensitivity of Boolean functions, and canalizing degrees. The impact of node mutations is assessed using as target measure the average Hamming distance between a non-mutated/wild-type network and a mutated network.

**Results:**

We find that for a biochemical signal transduction network consisting of several main signaling pathways whose nodes represent signaling molecules (mainly proteins), the algebraic method provides a robust classification of attribute contributions. This method indicates that for the biochemical network, the most significant impact is generated mainly by the combined effects of two attributes: out-degree, and average sensitivity of nodes.

**Conclusions:**

The results support the idea that both topological and dynamical properties of the nodes need to be under consideration. The algebraic method is robust against the choice of initial conditions and partition of data sets in training and testing sets for estimation of the nonadditive set functions of the information fusion procedure.

## 1 Background

Intracellular signaling pathways are important for the development and survival of living organisms, and many human diseases such as autoimmunity, cancer, diabetes and heart disease arise from aberrations in signaling [1]-[4]. In addition to the importance of intracellular signaling pathways, the last two decades of biomedical research have revealed their complex network-like structure [5]. Traditionally, signal transduction has been described as discrete linear pathways connecting cell surface receptors to cellular effectors, and through which information is transmitted and certain cellular responses are generated. However, individual pathways are highly interconnected with each other, involving extensive cross-talks and feedback loops; thus propagation of cellular signals does not necessarily occur in a linear fashion [6]. This complexity has been a major challenge in cell biology and an obstacle for the development of more effective therapeutic treatment for diseases such as cancer. To tackle this challenge, systems biology approach has started attracting a great deal of attention through the development of mathematical models of biological systems by combining experimental, theoretical and computational techniques.

In this paper, we use a previously published Boolean model of signal transduction in a generic cell to investigate the sensitivity of the network to molecule perturbations [7]. In Boolean networks the node activity can be described by two states, 1 and 0, active and inactive, and each node is updated based on logical relationships with other nodes. Boolean networks can model a variety of real or artificial networks such as signal transduction networks [7]-[15], gene regulatory networks [16]-[21], or neural networks [22]. There are several network/node attributes such as connectivity and types of Boolean functions (e.g., canalizing) that are responsible for the dynamics of Boolean networks [23],[24]. However, each of these individual attributes can be affected by any of the others. Thus, a true understanding of how these attributes combine to produce dynamical effects can only come from studying them in an integrated fashion.

The overall goal of this paper is to use a mathematical approach to *fuse* these attributes and determine which combination of attributes affects most the dynamics of the network. So far, research has focused mostly on simplified views involving networks that obey either one single category of Boolean functions, or are constructed using a fixed connectivity for all nodes or a widely used connectivity distribution [25],[26]. Little work has been done on assessing which of the attributes of the nodes has greater impact on the overall dynamics of the network. We show that a network’s sensitivity to perturbations is the result of combinations of certain attributes of the nodes in various degrees using a mathematical method based on a nonadditive set functions and nonlinear integrals, in particular the so-called Choquet integral [27]-[31].

It is intuitive that both topological and dynamical features have to be considered to understand sensitivity, and that has been illustrated in the literature under various network scenarios, e.g. [25] or [26]. Our work adds to that conversation and uses the nonadditive set function approach to go one step further and identify the joint node attributes that are the most important in assessing the sensitivity of the network to molecule perturbations. In particular we find that out of four topological and three dynamical node attributes under consideration, the out-degree of a node and the average sensitivity of the Boolean function governing the dynamics of the node have the most significant joint impact on the overall dynamics of the network and its sensitivity to individual node mutations. We provide an analysis of this result and how it compares to the roles of the other attributes under consideration.

The paper’s structure is as follows. In Section ‘Network, node attributes, and target measure’ we present the important aspects of the network under consideration and introduce the main node attributes used in the nonadditive set function method. We also introduce the target measure for the impact of node mutations on the dynamics of the network in order to compute the nonadditive set functions of the information fusion method. More mathematical information on the attributes and the target measure are presented in Section ‘Methods’, together with the basic mathematical background for the information fusion method and procedure for generating the nonadditive set functions from a given data set. We end Section ‘Methods’ with a description of the data sets and the approach used for our numerical investigations. The method is validated in Section ‘Results and discussion’ and the best combination of attributes is identified. An analysis of the results is also included. We finalize with conclusions and ideas for future research in Section ‘Conclusions’.

## 2 Network, node attributes, and target measure

We start this section by describing the network under consideration, followed by an overview of the node attributes used in information fusion, and the definition of the main target/quantity used to measure the impact of node mutations on the dynamics of the network in the mathematical procedure that is introduced in Section ‘Information fusion’.

The signal transduction network of a generic fibroblast cell considered in this paper consists of several main signaling pathways, including the receptor tyrosine kinase, the G-protein coupled receptor, and the integrin signaling pathway. A Boolean representation of this network has been provided in [7], and has been studied further in [32]. Furthermore, the fully annotated signal transduction model is freely available for simulations and/or download via the Cell Collective software from www.thecellcollective.org[33],[34]. Each node in the model represents a signaling molecule (mainly protein). The model also contains nine external input nodes which represent extracellular stimuli, adding a stochastic component to the network, or a “background noise” that exists in all biological systems.

We consider the following node attributes: *x*_1_: In-degree (connectivity). *x*_2_: Out-degree (number of downstream nodes). *x*_3_: Minimum path length (minimum number of edges/links from one of the nine external inputs). *x*_4_: Average path length (average [minimal] number of edges from the external inputs). *x*_5_: Bias (probability that the output of its Boolean function is a 1). *x*_6_: Average sensitivity of the Boolean function, which measures the likelihood that a single flip in an input vector generates a flip of the output of the Boolean function (See Section ‘Methods’ for more details). *x*_7_: Canalizing degree, which is a measure of the number of ways canalization occurs in the Boolean functions (See Section ‘Methods’ for more details).

The attributes considered in this paper represent only a few topological and dynamical node attributes that one could consider. We have selected them due to being very common in analyses of Boolean networks, keeping in mind also that our data-set has a fixed size that does not allow us to consider more than a few attributes at a time (to be explained in what follows). Let us discuss them briefly pointing out relationships between them in relation to the sensitivity of the nodes to mutations and noise in general. The in-degree is the typical parameter used in basically any Boolean network model and indicates the level of connectivity of a node. The in-degree distribution can distinguish between say random networks and scale-free networks. A large connectivity may be related to an increased sensitivity to changes in the inputs. A related attribute is the out-degree as a measure of the spread of influence of a given node. The more outgoing links a node has, the wider the spread of its influence, so a change in its state may have more impact on the other nodes. In the fibroblast network we have external stimuli that generate a “background noise” that exists in all biological systems as described in [7]. One could expect that the behavior of nodes with a shorter path to the cell surface be more susceptible to the extracellular noise. Therefore we consider also the minimum and average path length to the cell surface. A network with large connectivity and/or out-degree is expected to have smaller average path length. In general the average path length in a network is an indicator of a small-world property. However, one cannot separate the topological aspects from the dynamical attributes. For example, if the Boolean function of a node with a path length of one is the logical copy function (bias 0.5), then the output of this node is the same as the input received from the extracellular stimulus. Therefore, if the extracellular stimulus is random, then the behavior of the node is also random. However, if a node with a path length of one obeys a canalizing function, or a function with a bias close to 0 or 1, then the external noise can be to some extent filtered out. As a result, the behavior of the node can be rather predictable. On the other hand, large path lengths may diminish the sensitivity of the network to perturbations. Thus it is important to consider also dynamical attributes such as bias and canalizing degree of the Boolean functions, together with the topological attributes. A large or small bias may induce more order or canalization in the network, thus leading to less sensitivity to disturbances; while a bias close to 0.5 induces more randomness in the network behavior. Reduced sensitivity can result also from an increased degree of canalization. The average sensitivity of a Boolean function is a well studied complexity measure of how sensitive the output of the function is to changes in the inputs. It is clearly influenced by the bias, the connectivity, or the degree of canalization. This is a very natural choice of attribute when we are interested in assessing the impact of noise on the dynamics of the network. On the other hand, a small connectivity may generate more canalization in the network. Thus the attributes, either topological or dynamical, may be closely related and one cannot completely separate them in any analysis. Further related attributes may be considered in the future, such as clustering coefficients or depth of canalization for fully or partially nested canalizing functions. However, those are also related to the previously described attributes, so in this study, we take into account only seven basic attributes described in this paragraph and presented further in the next sections. This way we cover all the most basic attributes, both topological and dynamical, without increasing the number of attributes that may be very closely related, and allowing us to stay within the limits of our data-set.

We create a data set of all these attributes for each node, and fuse this information using nonlinear integrals with respect to nonadditive set functions and establish a target measure that represents the impact of node mutations on the dynamics of the network. We consider both activating and inactivating mutations, representing constitutively active nodes (e.g., gain-of-function mutations) and loss-of-function (or knock-out), respectively. Both of these scenarios are relevant to and common in many diseases, including cancer. One of early examples of an activating mutation includes the protein Ras which has been found to be constitutively active due to a mutation in the corresponding gene. This mutation can result in a continuous stimulation of cell growth and, consequently, in cancer.

**Figure 1:**
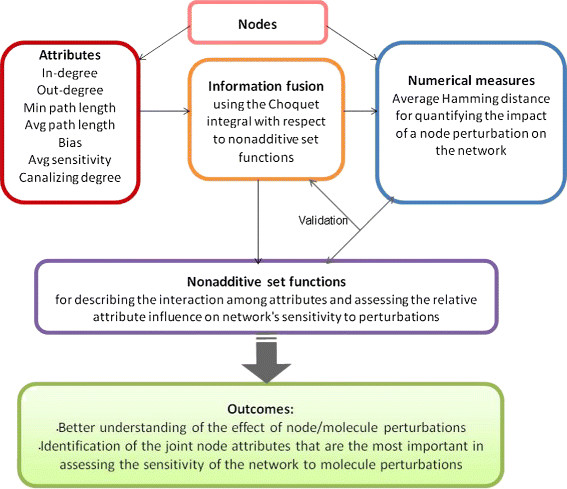
**Information fusion scheme.** Schematic description of the procedure used for the information fusion of node attributes and related outcomes.

The target value we consider in this work is the *average Hamming distance, AHD*, between a non-mutated/wild-type network and a mutated network (See Section ‘Methods’ for more details). The AHD will be computed under individual node mutations for the mathematical procedure described in what follows and depicted in the scheme of Figure 1.

We end this section with a note on network updating schemes. Logical networks could be updated according to different update schemes that may generate disturbances or noise in the network. Real networks such as genetical or biological, physical, neural, chemical, or social, are subject to regular disturbances and have the ability to reach functional diversity and aim to maintain the same state under environmental noise (e.g. food source or energy changes). Intrinsic or environmental disturbances can be modeled using asynchrony by updating the value of certain nodes in the network at different time steps (according to a deterministic or stochastic rule). Our goal is to focus on possible mutations within the network (e.g. genetic mutations) which can be modeled by freezing the value of a node over time, and we choose a synchronous network in which all nodes update their values at each time step, thus controlling the possible environmental disturbances.

## 3 Methods

### 3.1 Attributes

The average sensitivity *x*_6_ measures the likelihood that a single flip in an input vector generates a flip of the output of the Boolean function. More precisely, let *g*:{0,1}^*k*^→{0,1} be a Boolean function with *k* inputs, and *s*(*g*,*a*) the number of elements *b*∈{0,1}^*k*^ such that $H(a,b)=\overset{k}{\underset{j=1}{\sum }}|a_{j}-b_{j}|=1$ and *g*(*a*)≠*g*(*b*). The quantity *H*(*a*,*b*) is the Hamming distance between the vectors *a* and *b*. The average sensitivity of *g* is defined as the average of all the quantities *s*(*g*,*a*) with respect to a probability distribution of the input vectors *a*, so *avs*(*g*):=<*s*(*g*,*a*)>[26],[35]. In [26] it is shown that the average sensitivity is a suitable indicator of the dynamical behavior of a random Boolean network, whose value can reflect the regime in which the network operates. In this paper we consider a uniform probability distribution of the input vectors.

The canalizing degree *x*_7_ is a measure of canalizing inputs in the functions. Now, a Boolean function *g*:{0,1}^*k*^→{0,1} is canalizing if at least for one value of one of the inputs the output is fixed, irrespective of the values of the other inputs. Let *w* be the number of ways canalization occurs for a given Boolean function with *k* inputs, so that *w*∈{0,1,…,*k*,2*k*}. For example, in case of the logical AND function all inputs are canalizing with canalizing value 0 and canalized output 0, so that *w*=*k*. On the other hand, a constant function has *w*=2*k* which is the largest possible value for *w*. The canalizing degree of a node, *x*_7_, is the quotient *w*/(2*k*)∈ [ 0,1]. Canalizing functions may induce stability in certain genetic networks [24]. The nodes of the fibroblast network model [7] obey various Boolean rules including canalizing functions with one or more canalizing inputs, or nested/partially nested canalizing functions [32].

### 3.2 Target value

The target value is the average Hamming distance (AHD) between a non-muted/wild-type network and a mutated network. More precisely, let *a*(*t*)=(*a*_1_(*t*), *a*_2_(*t*),…,*a*_*N*_(*t*))∈{0,1}^*N*^ be the state of the network at time *t*. Then {*a*(0),*a*(1),…,*a*(*T*)} is the trajectory of the network with initial state *a*(0) up to time *T*. Consider now the trajectory of the same network under the activation of node *j*, that is {*b*(0),*b*(1),…,*b*(*t*),… } where *b*(0)=(*a*_1_(0),…*a*_*j*−1_(0),1,*a*_*j*+1_(0),…,*a*_*N*_(0)), and *b*_*j*_(*t*)=1 for all *t*. For inactivation we replace 1 by 0. Then

(1) $$\text{AHD}=\frac{1}{N\cdot T}\overset{N}{\underset{i=1}{\sum }}\overset{T}{\underset{t=1}{\sum }}\left|a_{i}(t)-b_{i}(t)\right|$$

### 3.3 Information fusion

This section contains a brief overview of the mathematical method used for information fusion based on nonadditive set functions and nonlinear integrals. The process of merging separate information sources using an aggregation tool into a one-dimensional datum on which we can make a prediction or classification decision, is called information fusion. Each information source in a data set is called an attribute and is denoted by *x*_*i*_,*i*=1,2,…,*n* (*n*=7 in our case). The numerical information obtained from *x*_*i*_ is an observation labelled *f*(*x*_*i*_), and *y* denotes a numerical evaluation for the target (AHD). To aggregate the numerical information obtained from various attributes, considering the interaction among the contribution rates of the attributes towards the aggregation target, a nonadditive set function defined on the power set of all aggregated attributes and a relevant nonlinear integral, such as the Choquet integral, are needed to replace the classical additive measure and the Lebesgue integral respectively [28]-[31]. The amount of combined contributions of various attributes toward the target may not equal the sum of individual contributions, since there may be a synergistic effect of the attributes toward the target. The nonadditive set functions (labeled *μ*) describe such interactions among attributes and model their relative importance.

We provide the description of the Choquet integral with respect to non-monotone nonadditive set functions used for this study. Let *X*={*x*_1_,*x*_2_,…,*x*_*n*_} be a nonempty finite set of attributes and *P*(*X*) be the power set of *X*. The nonadditive set function *μ* is a mapping from *P*(*X*) to [0,*∞*) where *μ*(*∅*)=0. Let *f*:*X*→(−*∞*,*∞*) be a measurable function on the measure space (*X*,*F*,*μ*), whose outputs represent the observed target values. The Choquet integral of *f* with respect to *μ* on *X* is defined as

(2) $$(C)\int f\;\mathrm{d\mu}=\overset{0}{\underset{-\infty }{\int }}[\mu (F_{\alpha })-\mu (X)]\mathrm{d\alpha}+\overset{\infty }{\underset{0}{\int }}\mu (F_{\alpha })\mathrm{d\alpha}$$

where *F*_*α*_={*x*| *f*(*x*)≥*α*} for *α*∈(−*∞*,*∞*), if both terms on the right-hand side of the formula exist, and not both are infinite.

To incorporate the cases where a set of attributes has a negative contribution toward the target we use a signed nonadditive set function *μ*:*P*(*X*)→(−*∞*,*∞*). When *μ* is a signed nonadditive set function, *μ* can be expressed as a difference of two nonadditive set functions *μ*^+^ and *μ*^−^:*μ*=*μ*^+^−*μ*^−^. Then the Choquet integral with respect to a signed nonadditive set function is defined as

(3) $$(C)\int f\;\mathrm{d\mu}=(C)\int f\;d\mu^{+}-(C)\int f\;d\mu^{-}.$$

This formula can be alternatively written as

(4) $$(C)\int f\;\mathrm{d\mu}=\overset{2^{n}-1}{\underset{j=1}{\sum }}z_{j}\mu_{j}$$

where $\mu_{j}=\mu \left(\underset{i:j_{i}=1}{⋃}\{x_{i}\}\right)=\mu^{+}\left(\underset{i:j_{i}=1}{⋃}\{x_{i}\}\right)-\mu^{-}\left(\underset{i:j_{i}=1}{⋃}\{x_{i}\}\right)$ if *j* is expressed in terms of binary digits *j*_*n*_,*j*_*n*−1_,…,*j*_1_ for every *j* and

(5) $$z_{j}=\underset{i:\text{frc}(j/2^{i})\in [1/2,1)}{\min}f(x_{i})-\underset{i:\text{frc}(j/2^{i})\in [0,1/2)}{\max}f(x_{i})$$

if it is positive or *j*=2^*n*^−1, and *z*_*j*_=0 otherwise. Here *frc* stands for fractional part.

If *f*_*k*_(*x*_*j*_),*k*=1,2,…,*l*,*j*=1,2,…,*n* represent *l* observations of the *n* attributes, and *y*_*k*_,*k*=1,2,…,*l* are the *l* observations for a chosen target value, then let *Z*=[*z*_*kj*_],*M*=[*μ*_*j*_] and *Y*=[*y*_*k*_] be the corresponding matrices. Then a linear model with respect to the unknown parameters can be created by setting *ZM*=*Y*. Now the optimal values of *μ* can be calculated by solving for *M* such that $||\text{ZM}-Y||=\overset{l}{\underset{k=1}{\sum }}{\left((C)\int f_{k}\mathrm{d\mu}-y_{k}\right)}^{2}$ is minimal. The advantage of this alternative formula is that the value of the Choquet integral is expressed as a linear function of the values of *μ* so that the optimal values of *μ* can be obtained by using an algebraic method. The details of implementation and effectiveness of the algebraic method can be found in [27].

In order to use this algebraic approach, we generate a data set with all the values of the attributes *x*_1_,…,*x*_7_. This is basically a matrix with *N*=130 lines corresponding to the nodes of the network and *n*+1 columns corresponding to the *n*=7 attributes and one target value *y*. The matrix basically contains the information of Table 1, where *f*_*i*_(*x*_*j*_) is the value of the *j*-th attribute of the *i*-th node. The matrix is split in two parts: the first *T* lines represent the training set, while the remaining *L*=*N*−*T* lines represent the testing set. The training data set is used to identify the nonadditive set functions *μ*. These are used to calculate the value of the Choquet integral representing the estimated target values by using the numerical observations of attributes from the testing set. The estimates are then compared to the actual target values from the testing data set to assess the accuracy of the prediction.

**Table 1:** Data-set for information fusion

**Node**	***x***_**1**_	***x***_**2**_	**…**	***x***_***n***_	***y***
1	*f*_1_(*x*_1_)	*f*_1_(*x*_2_)	…	*f*_1_(*x*_*n*_)	*y*_1_
2	*f*_2_(*x*_1_)	*f*_2_(*x*_2_)	…	*f*_2_(*x*_*n*_)	*y*_2_
…	…	…	…	…	…
*T*	*f*_*T*_(*x*_1_)	*f*_*T*_(*x*_2_)	…	*f*_*T*_(*x*_*n*_)	*y*_*T*_
*T*+1	*f*_*T*+1_(*x*_1_)	*f*_*T*+1_(*x*_2_)	…	*f*_*T*+1_(*x*_*n*_)	*y*_*T*+1_
…	…	…	…	…	…
*N*	*f*_*N*_(*x*_1_)	*f*_*N*_(*x*_2_)	…	*f*_*N*_(*x*_*n*_)	*y*_*N*_

The first *T* lines correspond to the training set, while the remaining lines correspond to the testing set. The attribute values are labeled with *x*, while the target values are labeled with *y*.

### 3.4 Data sets and the numerical procedure

In this section we describe the data sets and the numerical procedure, which combined with the mathematical formulae from the previous section, allow us to compute the nonadditive set functions.

To obtain the target values AHD, we generate a wild-type network and a mutated network with a single mutated node. Both networks are iterated 800 time steps starting with the same initial states. After removing a transient part of length 300 time steps, we calculate AHD over the remaining 500 time steps according to [**Def. (**1**)**]. This procedure is repeated 130 times to account for all individual mutations in the network. Furthermore, we also consider 100 different initial states of the network for each of the mutations above. In our previous analysis of a Boolean network model for the fibroblast network considered in this work [32], we noticed that the different types of Boolean rules governing the node dynamics lead to similar equilibria when modifying some of the parameters. These rules include various canalizing functions, threshold functions, and bias functions. The functions are classified in five large categories based on their impact on the activity level of the network (e.g. inhibitory or activating); this is done by plotting the activity level of the network, also known as the density of ones, at consecutive time points (Figure six in [32]). However, bifurcation diagrams along connectivity levels or proportions of these classes in the entire network indicate stability with a single fixed point, whose value depends on the modified parameters; the fixed points are reached in less than 200 time steps. In our current simulations we use 300 time steps to reach the attractors. Thus, in the context of the signal transduction network under consideration, previous research indicates stability of the dynamics with a frozen state in the long run, regardless of the initial state (when modifying connectivity and proportions as specified above). This behavior has been previously observed in simulations of the actual network using Cell Collective [34]. Therefore, for the purpose of this work, we find it reasonable to choose a fairly small number of initial conditions from the entire state space to ease the computational burden. As specified in [32], nodes belonging to a given class of Boolean functions may have a common behavior in the network. For example, both proteins Grb2 and Nck belong to the same class in that paper, and they are both adaptor proteins (that is they are accessory to main proteins in a signal transduction pathway mediating specific protein-protein interactions that drive the formation of protein complexes). By applying perturbations to various types of nodes or classes of nodes we may be able to associate biological and mathematical aspects of a particular class of nodes which may not be observed in laboratory. For more information we refer the reader to [34] and [32].

**Figure 2:**
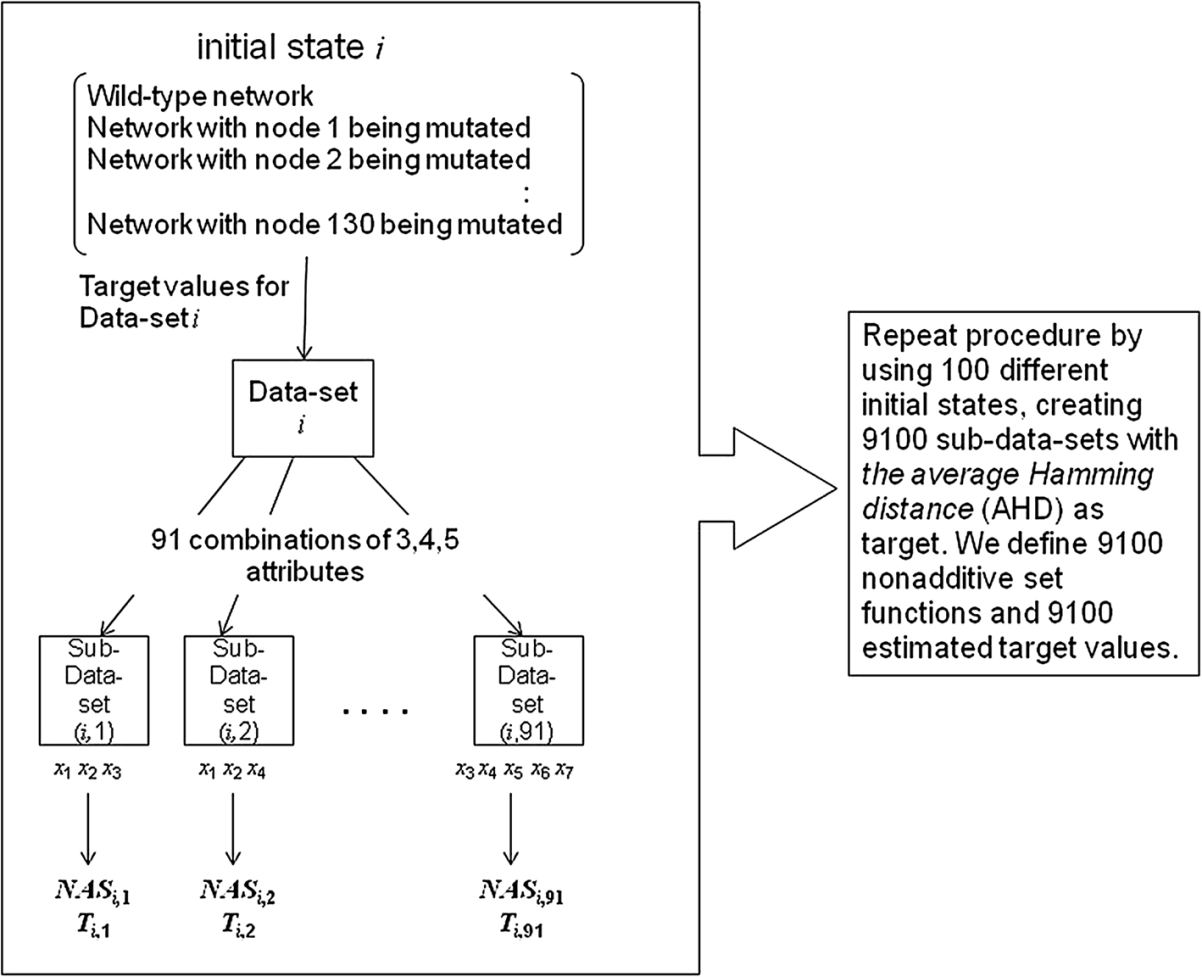
**Creation of sub-data-sets.***NAS*_*i*,*j*_,*i*=1,2,…100,*j*=1,2,…91, stands for the nonadditive set function obtained from sub-data-set (*i*,*j*). *T*_*i*,*j*_ stands for the estimated target values obtained from sub-data-set (*i*,*j*).

**Figure 3:**
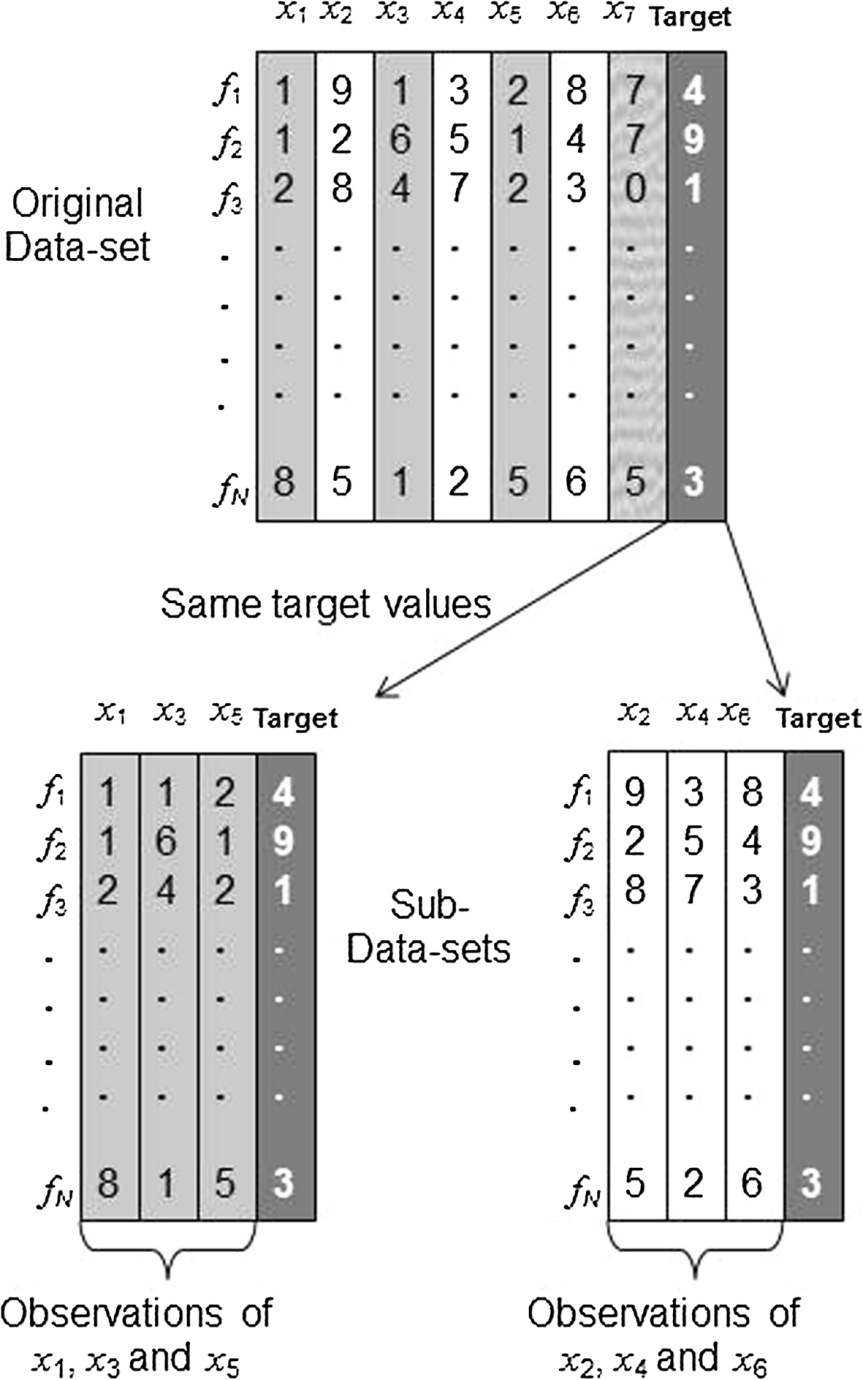
**Simplified sub-data-sets.** A simplified example of a data-set and sub-data-sets.

Each data set with 130 lines is split into the training set and the testing set. The size of the training set must be at least 2^*n*^−1 where *n* is the number of attributes. Also according to our previous studies [27], the prediction is more accurate when the size of a training set is much larger than the minimum size of a training set. Therefore, we have to combine less than seven attributes at a time. We choose to use *n*=3,4,5 attributes at a time to define nonadditive set functions, so that both the training and testing data sets are large enough. Since seven attributes of each node are considered, there are ∑i=357i=91 combinations of 3, 4 and 5 attributes. Note that combinations of fewer than three attributes are automatically considered in the mathematical approach even if *n*≥3, and repetitions of combinations occur as *n* is increased since for each *n* we consider all the possible combinations of 1,2,…*n* attributes. By varying *n* we can make sure that the method works well even with less data for testing and training. Thus we test our method with three different training sets whose size increases with larger values of *n*. By using each of these 91 combinations of attributes, we create sub-data-sets (i.e. using the same target values with different combinations of attributes) and apply the algebraic method to each of them as portrayed in Figure 2. Thus we define nonadditive set functions and estimate the values of the Choquet integral from 9100 sub-data-sets using AHD as target. Figure 3 gives a simplified example of sub-data-sets created from an original sub-data set for more clarity. We use the nonadditive set functions estimated from the training set to generate estimated target values for the testing set. Then we compare these estimates to the real target values computed for the testing set from the original data. If they are close enough (to be defined in the next section) then the method is validated and we can use it to identify the best combination of attributes. The validation and analysis of the results based on the above procedure unfolds in several steps as described next.

### 3.5 Average error of target values

The average error (over all *i*=1,2,…,100 initial states) between the estimated target values and the original target values from the testing set is calculated as follows: consider the *j*-th combination of attributes, where *j*=1,2,…,91, corresponding to the combination of *n*=3,4 or 5 attributes as indicated in Section ‘Methods’. Let *L* be the length of the testing set; in case the training set has length 120, we get *L*=10. For each *l*=1,2,…,*L* (these correspond to lines *T*+1,…,*N* of Table 1), denote by $y_{i,j}^{l}$ the *l*-th target value obtained from the testing set for the sub-data-sets (*i*,*j*), and ${ŷ}_{i,j}^{\;l}$ is the corresponding estimated target value obtained with the information fusion procedure. In the simulations the values of the nonadditive functions are computed by applying a Matlab numerical procedure for [**Eq. (**2**)**]. Then the quantity

(6) $$\;Y_{i,j}=\frac{1}{L}\overset{L}{\underset{l=1}{\sum }}|y_{i,j}^{l}-{ŷ}_{i,j}^{l}|,\;\;\;i=1,2,\ldots ,100,\;j=1,2,\ldots ,91$$

is the average error of the estimates over the testing set for the sub-data-set (*i*,*j*). Then we average these quantities over all the initial conditions to obtain the average error of the *j*-th combination of attributes, namely

(7) $$E_{j}=\frac{1}{100}\overset{100}{\underset{i=1}{\sum }}Y_{i,j},$$

which we use to determine the accuracy of the method. The smaller the average error, the better the estimates.

Before we present our results, let us review briefly the main properties and hypotheses used for simulations. The network under consideration of 130 nodes is governed by Boolean functions of various types (including threshold, canalizing, and bias), and is updated synchronously. The network is quenched, therefore the inputs and the Boolean rules are fixed throughout the evolution of the network as in the signal transduction model [7]. The information fusion procedure is based on the seven attributes described previously: in-degree, out-degree, minimum path length, average path length, bias, average sensitivity, and canalizing degree. To assess the optimal non-linear combination of attributes we compute the nonadditive set functions *μ*, and using them, we determine the impact of individual mutations, both activating and inactivating, by computing the AHD as in [**Eq. (**1**)**]. We use these values in the results section to follow.

## 4 Results and discussion

It is important to validate our approach. More precisely, once the nonadditive set functions are estimated from the training set (chosen of length 120 out of the available 130), they are used to generate AHD values for the data in the testing set. These estimated AHD values are then directly compared to the original AHD values of the testing set that are obtained with the actual data. If the error of the estimation is small, then we accept the estimates as valid. This validation is presented in Section ‘Validation of the method’. Once the method is validated, we can find the best combination of attributes by basically identifying the nonadditive set functions that minimize the error. Besides the simple error term used in validation we incorporate also an error term that insures consistency among the initial network states used in simulations. The results of this approach are presented in Section ‘Best combination of attributes’. At the same time we want to make sure the estimates are not dependent on the choice of training and testing sets by applying the method several times with modified training and testing sets. We modify the length as well as the content of these sets to validate further the proposed method. We also add an extra refinement to the choice of the best combination of attributes by choosing the (minimal error) combination of attributes that leads to the highest values of the nonadditive set functions. This is done in Section ‘Five-fold cross-validation’. We finalize in Section ‘Analysis and discussion of combinations of attributes’ with an analysis of the relation between attributes based on all nonadditive set functions for all the combinations of attributes that have been used in this work, not only the best combination.

### 4.1 Validation of the method

We define nonadditive set functions by using the training set of size 120 out of the available 130 since with a bigger training set it is more likely to capture a limited effect of node perturbation. As specified in Section ‘Methods’, the choice of *n*≤5 insures that the algebraic procedure is meaningful even for a data set of 130 nodes. On the other hand, we will use what we call a five-fold cross-validation procedure to show that the estimates for the nonadditive functions are not dependent on the choice of training and testing sets.

**Figure 4:**
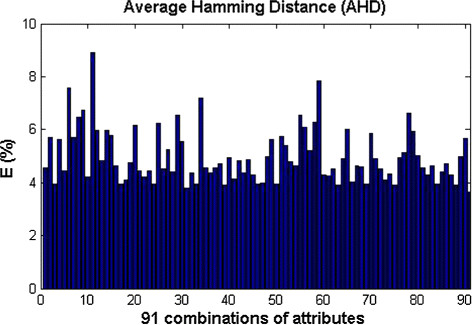
**Average error of estimated target values.** The *x*−axis represents all the 91 combinations of 3, 4 and 5 attributes, while the *y*-axis represents the average error between the original target values obtained from the testing sets and the estimated target values for activating mutations. Notice the rather low error values.

Once the data are divided into training versus testing sets we find the average error (over all *i*=1,2,…,100 initial states) between the estimated target values and the original target values from the testing set. The smaller the average error, the better the estimates (See Section ‘Methods’ for more details). As shown in Figure 4 for activating mutations, the average error between the original and the estimated target values have small magnitudes. Thus, the estimated nonadditive set functions can capture the interaction among attributes and therefore can be used to model their relative importance. Similar results are obtained for inactivating mutations.

We have also checked the additivity of the estimated set functions. If *X*={*x*_1_,*x*_2_,…,*x*_*n*_} is a set of *n* attributes and for a given set function *μ* we have that $\overset{n}{\underset{i=1}{\sum }}\mu (\{x_{i}\})\neq \mu (X)$, then the set function is not additive. Our computations indicate that for all the 9100 set functions this condition is met, so they are indeed nonadditive. This justifies the use of the Choquet integral as an aggregation tool in the information fusion of the node attributes. Thus the fact that the joint contribution of the attributes has more impact on the targets than the stand alone attributes is taken into account. More precisely, for each combination of attributes we compare the magnitude of the corresponding *μ* values. For example, a scenario of only three attributes, say {*x*_1_,*x*_2_,*x*_3_}, yields *μ*({*x*_1_}), *μ*({*x*_2_}), *μ*({*x*_3_}), *μ*({*x*_1_,*x*_2_}), *μ*({*x*_1_,*x*_3_}), *μ*({*x*_2_,*x*_3_}), *μ*({*x*_1_,*x*_2_,*x*_3_}). We note that in most cases, the maximum *μ* of all these values corresponds to a combination of attributes, rather than a single attribute. For example in case of activating mutations, for 82.4 *%* (AHD) of all combinations of attributes, the maximum *μ* value corresponds to combinations of at least two attributes. Thus combinations of various attributes of nodes may have an increased influence on the network sensitivity to node perturbation. We can now apply the procedure to define the best combination of attributes and from that combination find which combination of attributes yields the most significant sensitivity to mutations.

### 4.2 Best combination of attributes

Now we can find the best combination of attributes by basically identifying the nonadditive set functions that minimize the error. However we need to make sure that our method is also consistent across the initial network states used in simulations before we decide what is the best combination.

We consider a numerical measure that can identify the combination of attributes that has the most impact on the sensitivity of the network to mutations. Aside from minimizing the error in [**Eq. (**4**)**], we want our estimates to be fairly consistent across the 100 random initial conditions for which we obtain 100 different values for average errors and nonadditive set functions. Therefore, we consider two other quantities besides the average error in [**Eq. (**4**)**]: the standard deviation of the errors (or consistency in target values, CT) and the standard deviation of the estimated nonadditive set functions (or consistency in nonadditive set functions, CM), with the goal of finding the combination of attributes that minimizes the variation due to the choice of the initial states.

The consistency in target values is given by

(8) $$CT_{j}=\text{std}{\left[Y_{i,j}\right]}_{i},\;j=1,2,\ldots 91$$

where *Y*_*i*,*j*_ are given in [**Eq. (**3**)**], and *std*[ ·]_*i*_ stands for standard deviation over the 100 different initial states.

To define the consistency in nonadditive set functions we first consider the quantities

(9) $$\;S_{i_{1}i_{2}\ldots i_{m}}=\text{std}{\left[\mu (\{x_{i_{1}},x_{i_{2}},\ldots ,x_{i_{m}}\})\right]}_{i},\;m=1,2,\ldots n$$

where the indices 1<=*i*_1_<=*i*_2_<=⋯<=*i*_*m*_<=*n* identify the collection of attributes for which we compute the nonadditive set function, and *n* is the number of attributes in the *j*th combination of attributes. For a given *n* there are 2^*n*^−1 such collections of indices. Then we average the quantities $S_{i_{1}i_{2}\ldots i_{m}}$ to obtain

(10) $$CM_{j}=\frac{1}{2^{n}-1}\underset{i_{1},i_{2},\ldots ,i_{m}}{\sum }\;S_{i_{1}i_{2}\ldots i_{m}}$$

Thus, for the consistency in nonadditive set functions, we average the individual standard deviations over all possible values of nonadditive set functions.

We define the *best combination of attributes* from among the given *n* attributes to be the one that minimizes the norm of the vector (*E*_*j*_,*CT*_*j*_,*CM*_*j*_). Thus, the index *J*∈{1,2,…,91} of the *best combination of attributes* is given by

(11) $$J={\arg\min}_{j}\left[\sqrt{E_{j}^{2}+CT_{j}^{2}+CM_{j}^{2}}\right]$$

A fairly small norm insures that all three individual measures are small enough. Formula (7) represents our choice of measure for identifying the best combination of attributes in this paper. Alternative measures may be considered, however we have no basis for any speculation on the effect of the chosen measure. Our results reflect the measure defined in (7).

**Figure 5:**
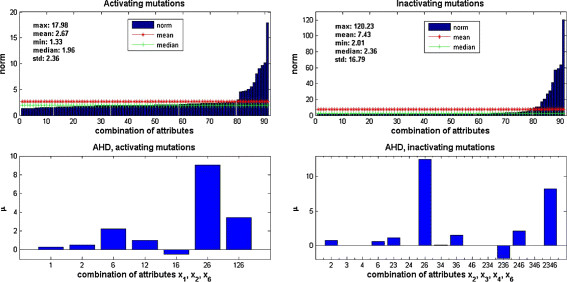
**Norm values and nonadditive set functions.** Top: The x-axis represents a total of 91 combinations of attributes sorted according to their associated norm in [**Eq. (**7**)**]. The far left value corresponds to the best combination of attributes: *x*_1_ (in-degree), *x*_2_ (out-degree), and *x*_6_ (average sensitivity) for activating mutations, and *x*_2_, *x*_3_ (minimum path length), *x*_4_ (average path length), and *x*_6_ for inactivating mutations. The norms are generally lower for activating mutations. Bottom: The nonadditive set functions obtained for AHD and in/activating mutations. The x-labels indicate the combinations that yield each of the bars of the graphs; for example 246 means the combination of *x*_2_,*x*_4_,*x*_6_. The largest *μ* value is given by *x*_2_ (out-degree) and *x*_6_ (average sensitivity).

Figure 5 (top plots) shows the result for all 91 combinations of attributes sorted in ascending order. The best combination of attributes corresponding to the minimum norm (far left) is *x*_1_ (in-degree), *x*_2_ (out-degree), and *x*_6_ (average sensitivity of Boolean functions) for activating mutations, and *x*_2_, *x*_3_ (minimum path length), *x*_4_ (average path length), and *x*_6_ for inactivating mutations. For each of these we will select the combination that leads to the highest *μ* values.

### 4.3 Five-fold cross-validation

The results obtained in the previous section are based on finding nonadditive set functions from a training set of size 120 out of the available 130, so the testing set is of size 10. In this section we show that the estimates are not dependent on the choice of training and testing sets. For this purpose we modify the training and testing sets in length as well as in content. This process is described next. We also refine the choice of the best combination of attributes by choosing the one that leads to the highest *μ* values. The choice is made from the minimal error combinations identified in the previous section.

We randomly partition the 100 data sets corresponding to the best combination of attributes into five subsets that are alternatively used as training versus testing sets. One subset of length 26 is used for testing while the remaining subsets of length 104 for training. This process is repeated five times with a different subset for testing. This method has the advantage that every observation from the data set is used only once for testing and training. Going beyond five repetitions does not generate significant improvements.

We obtain a total of seven/fifteen values *μ* for activating/inactivating mutations, together with the average error for each of the five testing/training sets. We calculate the overall mean of these average errors and the average of the *μ* values over the 100 initial conditions. The overall mean of the average errors is 7.53*%*. Although there is a slight increase in the average errors in comparison to those obtained with the training data of size of 120, the errors are still quite small.

We also use different sizes of training and testing sets to investigate their impact on the accuracy of the average error changes and if there is a risk of under-testing when we use the five-fold cross-validation method. The results are shown in Table 2 for AHD under activating mutations. In both cases, the average errors drop as the training data size increases. However, the drop is very small once a certain threshold is reached. For example, when the training set represents 50% of the data, the average error is almost as small as for 80% of the data, thus supporting the results obtained by the five-fold cross-validation method.

**Table 2:** AHD error

**Training data size**	**Testing data size**	**Average error AHD (*%*)**
65 (50 *%*)	65	8.65
75 (57.7 *%*)	55	8.06
85 (65.4 *%*)	45	8.15
95 (73.1 *%*)	35	7.72
105 (80.8 *%*)	25	7.81
115 (88.5 *%*)	15	6.92

The impact of increased training set size on the average error for AHD under activating mutations is presented as a percentage.

In Figure 5 (bottom plots) we graph the values of the nonadditive set functions obtained for the best combination {*x*_1_,*x*_2_,*x*_6_} for activating mutations, and {*x*_2_,*x*_3_,*x*_4_,*x*_6_} for inactivating mutations. As mentioned earlier, the magnitude of *μ* indicates the amount of contributions made by an attribute or combination of attributes toward the target. When the joint contribution of certain attributes is greater than the sum of an individual contribution of those attributes, there is a synergistic effect of those attributes toward the target. On the other hand, if the joint contribution of attributes is smaller, contributions made by those attributes toward the target are being offset [27]. As shown in Figure 5, the differences in the magnitude of *μ*’s except the greatest *μ* are relatively small. Note that the joint contribution of *x*_2_, the out-degree, and *x*_6_, the average sensitivity of the Boolean function of a node, is the greatest and significantly larger than all the others. This holds for either activating or inactivating mutations. In other words, the out-degree and the average sensitivity of Boolean function jointly serve as a good predictor for the impact on the network dynamics of a node mutation. We find that this result is not only limited to the best combination of attributes {*x*_1_,*x*_2_,*x*_6_}. There are 25 out of 91 combinations of attributes which include both *x*_2_ and *x*_6_. Actually, 40 *%* of these 25 combinations, include *x*_2_ and *x*_6_ in the combination with the greatest *μ*.

For a description of the whole method see the Additional file 1 where we provide a detailed algorithm, given step by step.

### 4.4 Analysis and discussion of combinations of attributes

Based on the results obtained from our mathematical method we can now discuss the relation between attributes with the help of the values of nonadditive set functions for all the combinations of attributes that have been used in this work. We compare these results with some other results in the literature. We go beyond the attributes that were find as the best combination to explore further possible relationships.

To make this analysis concise, we focus on activating mutations (although the same procedure was applied to inactivating mutations). In the literature, the in-degree of a node, *x*_1_, has been used as the main parameter for analyzing the network evolution. For example, by using the *NK* Boolean model proposed by Kauffman, Derrida and Pomeau obtained theoretical results showing that the phase transition is controlled by the in-degree and the bias of a node [23] in the context of the entire ensemble of *NK* networks. We emphasize again that both topological and dynamical parameters have to be considered for a suitable analysis of Boolean networks, even if there may be correlations between them. Our focus is on a numerical assessment (rather than a theoretical investigation) of a particular type of network with fixed links and Boolean functions throughout the evolution of the network that represents a sample of a signal transduction network. Thus our statistical assessment focuses not on an entire ensemble of network, but on a very specific one, so our results pertain to the particular network under consideration. In the future we plan on analyzing other related networks to identify potential commonalities and explore attributes that may be generally important for networks with certain characteristics.

In our simulations, 64*%* of all combinations of attributes which give the greatest *μ* values include *x*_2_ (the out-degree) while only 53*%* of combinations of attributes include *x*_1_ (in-degree). A mutated node with a large number of downstream nodes has potential for amplifying the effect of the mutation as it spreads to the rest of the network. However, the out-degree alone may not be the best predictor for the impact of mutations, since the actual Boolean function governing the dynamics of the node needs to be taken into account. This fact is confirmed by our result that *x*_2_ alone does not yield the greatest *μ* values. Observe in Figure 5 (bottom) that the joint nonadditive set functions have significantly larger values than the individual attributes. Also notice the negative impact of certain attributes.

Going beyond the attributes used in Figure 5 (bottom), there is also a possibility of the bias of a node, *x*_5_, being a good indicator for the impact of mutating a node on the network. In fact, *x*_5_ alone gives the greatest *μ* value for 12*%* of combinations of attributes which include *x*_5_. Furthermore, *x*_6_ (the average sensitivity of a Boolean function) and *x*_7_ (canalizing degree), which are bias-related, give the greatest *μ* value for 8*%* of combinations of attributes which include *x*_6_ and *x*_7_ respectively. None of the other attributes by themselves gives the greatest *μ* value.

Now let us briefly consider *x*_7_ (canalizing degree). If a mutated node has multiple canalizing variables that yield a canalized value 0, then the effect of mutation ON is greater because this node is supposed to have a lower probability of being active without mutation. Thus, the canalizing degree can indicate the effect of mutating a node to some extent, but it does not distinguish between canalized values 0 or 1, which matters if we are interested in activating versus inactivating mutations.

We have shown that the bias and bias related attributes have a potential of being good predictors of the impact of mutating a node. However, for 88*%* of combinations of attributes including *x*_5_ and 92*%* of those including *x*_6_ or *x*_7_, the attributes alone do not give the greatest *μ* value. When assessing the impact of mutating a node, we consider the gap between what is supposed to happen with and without mutation, together with how the effect of mutation spreads. That can happen either through a few downstream nodes or through a large number of downstream nodes. That is to say, the combination of attributes *x*_2_ with *x*_5_,*x*_6_ and/or *x*_7_ should be a good predictor for the impact of mutating a node on the network. This argument is supported by the result that 57*%* of combination of attributes which include *x*_2_ give the greatest *μ* value when *x*_2_ is combined with *x*_5_,*x*_6_ and/or *x*_7_. From this perspective, it is reasonable that the greatest *μ* value is obtained from the joint combination of out-degree and the average sensitivity of a Boolean function.

This work supplements previous research showing that it is important for network analyses in the area of drug target discovery to consider not only the *static* properties (e.g., in/out-degree, etc.) of individual nodes (e.g., genes or proteins) of the network (such as in [36]), but also properties that give rise to the underlying dynamics (e.g., bias and/or sensitivity). Logical models provide new opportunities to improve the predictive capabilities of computational models as they are easy to construct, and analyses via computer simulations are efficient and capable of covering a relatively large number conditions, and have also been applied to drug target prediction [12],[37], as well as prediction of potential drug side-effects and sensitivity [38].

## 5 Conclusions

In this paper, we apply a mathematical method of information fusion based on nonadditive set functions and the Choquet integral to a Boolean model of biochemical signal transduction. Nonadditive set functions are defined for all possible combinations of attributes and can be used to model their relative importance and interactions. We use an algebraic method to identify the nonadditive set functions and investigate which attribute or combination of attributes is important in predicting the sensitivity of the network to molecule perturbations. Our results support the hypothesis that combinations of different attributes of a node in various degrees play an important role in determining a network’s sensitivity to perturbations, more than the individual attributes. Specifically, we find that the out-degree of a node and the sensitivity of a Boolean function to perturbations have the most significant joint impact on the overall dynamics of the network and its sensitivity to perturbations. The method presented in this work is applicable to any network whose characteristics can be used to generate databases similar to those considered in this paper. Of course, the associated computational burden can restrict the network size that one could consider; however, large networks offer expanded training and testing sets which automatically improve the accuracy of the results. Our existing simulation tools are relatively efficient; for example using Cell Collective [33] we are currently able to easily simulate our largest model with approximately 600 nodes and more than 1,000 interactions. That model will be subject for future analysis using the algorithm of this paper. Also, Matlab, which was used for our information fusion procedure, is also capable of handling very large matrices.

To our knowledge the work in this paper is the first attempt to assess the importance of nonlinear combinations of attributes in a Boolean network. Previous studies on Boolean networks mostly assume several parameters as important from the very beginning (such as connectivity or bias) and use those to identify dynamics or robustness properties. On the other hand, the Choquet integral has already been used in the area of information fusion for other biological or non-biological systems, so it is a tool that has already shown its usefulness in different contexts. Therefore we believe that the information fusion procedure proposed in this paper has the ability to open the discussion on various aspects of the dynamics and robustness of Boolean networks using new non-linear tools from the area of information fusion.

The immediate continuation of this work would be to categorize the magnitude of the most significant attributes that lead to a certain magnitude of the sensitivity measures. Thus one can identify the types of molecules that could be targeted in therapies in order to generate a faster response to a disease treatment, and indicate in which way the attributes should be modified for this purpose.

Delving further into the mathematical aspect of the information fusion on Boolean networks and the usage of more advanced fusion techniques is one of our future goals. For instance, a convincing way for the existence of interaction of attributes is to take the Mobius transformation for the set function and to see whether its values at sets consisting of more than one attribute are significantly larger than zero. Similarly, one can improve the method by considering a linear or quadratic core of the Choquet integral.

On the other hand, we are also considering alternative target measures for the information fusion procedure. It might be important to look at attributes that capture biologically meaningful, possibly higher-level properties. For example, in [20] the authors use the mean first passage time from one state of the network to another (desired) state, to identify the best candidate genes for intervention in a gene regulatory network governed by probabilistic Boolean functions which are randomly chosen for each node at each time step. The signal transduction network considered in this paper is governed by Boolean rules fixed by the actual biological processes they represent. However, it would be of interest to consider the alternative target measure of mean passage time to identify the similarities and differences with the AHD approach. The similarities would point out some possible universal best attributes that are independent of the target measures, while the differences would allow one to adapt to the actual goal of the sensitivity analysis. This could be identifying the effects of individual mutations, versus say identifying the mutations that lead to a certain dynamical behavior in the shortest amount of time. At the same time, instead of comparing trajectories as in AHD it might also be useful to focus on reachability of biologically important attractors.

Future research could also explore how outcomes will be different if we include attributes not considered in this study, such as feedback loop or clustering information. We are also interested in trying different methods to preselect attributes to be used for the information fusion procedure instead of trying all combinations of attributes of a node in the network. Moreover, applying the method to an asynchronous network might reveal further aspects of the impact of intrinsic noise in the system in combination with node mutations. Our method is applicable to any type of update procedure.

At the same time, larger data sets of networks may provide more data for information fusion and results could become more accurate, since the training and testing sets would be expanded. However, large networks have huge state spaces. Therefore the sample of initial conditions needs to be chosen carefully. In general, it is reasonable to assume that the networks are non-ergodic, so not all states are equally likely at a given time point. Thus, by taking into account the long run activity of the network, it may be useful to consider sampling only the equilibrium state space or distribution, or a biologically meaningful wild-type behavior, thus severely restricting the state space. A similar approach has been recently used for finding the critical condition for the average sensitivity of a Boolean network model governed by nested and partially nested canalizing functions [39],[40].

Furthermore, exploring other biological networks could reveal some general attribute combinations that may be independent of the data set, thus identifying some underlying characteristics as universal predictors for the sensitivity to perturbations. We believe that the outcomes of these studies have the potential for providing a better understanding of the underlying mechanism of complex signaling networks as well as identifying possible target molecules and/or attributes for disease treatment.

## Competing interests

The authors declare that they have no competing interests.

## Authors’ contributions

NK, TH, and JAR conceived the biological background and approach, and provided the Boolean network data. NK and ZW conceived the information fusion method. NK and MTM conceived and performed the selection of attributes, the target measure, the validation, and the best attribute combination approach. NK conducted the analysis and performed most of the simulations. TH and LA performed some additional simulations and data analyses. NK, MTM, and TH wrote the manuscript. MTM, TH, JAR, ZW critically revised the manuscript. All authors read and approved the final manuscript.

## Additional file

## Supplementary Material

Additional file 1 Supplementary Material. Click here for file
