# Supplementary material for: Sensitivity analysis of biological Boolean networks using information fusion based on nonadditive set functions
Source: BMC Syst Biol. 2014 Sep 5;8:92. doi: 10.1186/s12918-014-0092-4 (PMC4363947; doi:10.1186/s12918-014-0092-4)
Supplement: Additional file 1 — Supplementary Material. [file s12918-014-0092-4-S1.pdf]

SENSITIVITY ANALYSIS OF BIOLOGICAL BOOLEAN NETWORKS USING INFORMATION FUSION BASED ON NONADDITIVE SET FUNCTIONS - SUPPLEMENTARY MATERIAL

NAOMI KOCHI[+], TOMÁŠ HELIKAR[†*], LAURA ALLEN[†], JIM A. ROGERS[†], ZHENYUAN WANG[†], AND MIHAELA TEODORA MATACHE[†]

[+]Department of Genetics, Cell Biology, and Anatomy
University of Nebraska Medical Center, Omaha NE 68198, USA

[†]Department of Mathematics
University of Nebraska at Omaha, Omaha, NE 68182, USA

[*]Department of Biochemistry
University of Nebraska-Lincoln, Lincoln, NE 68588, USA

In this supplementary material we provide the whole method described in a detailed algorithm, given step by step. We recall the elements and formulas that are essential for the algorithm.

Beginning of algorithm

Data-set: attribute values.

---

(1) Identify the attributes of interest. In this paper we use:
- x_1: In-degree (connectivity).
- x_2: Out-degree (number of downstream nodes).
- x_3: Minimum path length (minimum number of edges/links from one of the nine external inputs).
- x_4: Average path length (average [minimal] number of edges from the external inputs).
- x_5: Bias (probability that the output of its Boolean function is a 1).

TABLE 1. Additional file 1: Table S1. Data-set containing the values of all 7 attributes for each node.

Node	x_1	x_2	...	x_7
1	$f_1(x_1)$	$f_1(x_2)$	...	$f_1(x_7)$
2	$f_2(x_1)$	$f_2(x_2)$	...	$f_2(x_7)$
...	...	...	...	...
130	$f_{130}(x_1)$	$f_{130}(x_2)$	...	$f_{130}(x_7)$

- x_6: Average sensitivity of the Boolean function, which measures the likelihood that a single flip in an input. vector generates a flip of the output of the Boolean function.
- x_7: Canalizing degree which a measure of the number of ways canalization occurs in the Boolean functions.

(2) Collect the values of attributes for each node of the network in a matrix like Table 1. The fibroblast network has 130 nodes, so the the resulting matrix has size 130×7.

Iterations of the network for various initial states and mutations. Computation of the target values.

(3) Generate many initial states (we used 100 - see explanations in the main text):

FOR NumberInitialState $= 1, 2, \ldots 100$ perform the following steps:

(a) Select an initial state of the network $a(0) = (a_1(0), a_2(0), \ldots, a_{130}(0)) \in \{0, 1\}^{130}$ (the wild-type network).

(b) Generate the target values as follows:

FOR $j = 1, 2, \ldots 130$ perform the following steps:

i. Generate the mutated initial state $b(0) = (a_1(0), \ldots a_{j-1}(0), 1, a_{j+1}(0), \ldots, a_N(0))$ where the jth node is considered an activating mutation; for inactivation replace 1 by 0.

ii. Iterate both the wild-type and the mutated network for $T = 800$ time steps, using the Boolean rules governing the behavior of the individual nodes. The orbits are: $\{a(0), a(1), \ldots, a(800)\}$ and $\{b(0), b(1), \ldots, b(800)\}$ with $b_j(t) = 1$ for all time steps $t = 1, 2, \ldots, 800$.

Table 2. Additional file 1: Table S2. Data-set containing the values of all 7 attributes for each node together with the target values obtained for the given initial state.

Node	x_1	x_2	...	x_7	Target
1	$f_1(x_1)$	$f_1(x_2)$	...	$f_1(x_7)$	AHD(1,NumberInitialState)
2	$f_2(x_1)$	$f_2(x_2)$	...	$f_2(x_7)$	AHD(2,NumberInitialState)
...	...	...	...	...	...
130	$f_{130}(x_1)$	$f_{130}(x_2)$	...	$f_{130}(x_7)$	AHD(130,NumberInitialState)

iii. Compute the target value, AHD, over the last 500 iterations, according to the formula

$$AHD(j, NumberInitialState) = \frac{1}{130 \cdot 500} \sum_{i=1}^{130} \sum_{t=301}^{800} |a_i(t) - b_i(t)|$$

END FOR LOOP $j = 1, 2, \ldots 130$

(c) Extend the matrix of Table 1 with an extra column containing the 130 target values obtained from mutating the 130 nodes one by one. The extended data-set is shown in Table 2, and corresponds to the top table in Figure 3 of the main text.

Sub-data-sets.

---

(4) Choose $n = 3, 4, 5$ attributes at a time to define nonadditive set functions; this gives $\sum_{i=3}^{5} \binom{7}{i} = 91$ possible combinations out of 7 attributes. Create $91 \times 100 = 9100$ individual sub-data-sets corresponding to the 91 combinations of attributes and the 100 initial states. A sample sub-data-set is shown in Table 3 and corresponds to Figure 2 and the bottom of Figure 3 in the main text. Observe that the target values are the same (by nodes) for any combination of attributes in the sub-data-bases and a selected initial state. The information fusion can be performed using one single value of n; however in our work we use all three, so the steps for the estimation of the nonadditive set functions shown below are repeated for each n.

TABLE 3. Additional file 1: Table S3. Sample sub-data-set containing the values of 3 of the 7 attributes for each node together with the target values obtained for the given initial state.

Node	x_1	x_2	x_6	Target
1	$f_1(x_1)$	$f_1(x_2)$	$f_1(x_6)$	AHD(1,NumberInitialState)
2	$f_2(x_1)$	$f_2(x_2)$	$f_2(x_6)$	AHD(2,NumberInitialState)
...	...	...	...	...
130	$f_{130}(x_1)$	$f_{130}(x_2)$	$f_{130}(x_6)$	AHD(130,NumberInitialState)

Information fusion. Estimation of the nonadditive set functions and target values for the testing set.

---

(5) Calculate the nonadditive set functions for each of the 9100 sub-data-sets and generate estimated target values:

FOR NumberSubDataSet $= 1, 2, \dots 9100$ perform the following steps:

(a) Split the sub-data-set in two parts: the first $T \geq 2^n - 1$ lines represent the training set, used to identify the nonadditive set functions μ. Here n is the number of combined attributes. The remaining $L = 130 - T$ lines represent the testing set. We choose $T = 120$ since larger training sets produce more accurate estimations of the nonadditive set functions. In Section 4.3 of the main text (five-fold cross-validation) we discuss that a smaller training set produces only slightly less accurate estimates. Also, the choice of the T nodes to be used for training does not influence the outcome of the method. A basic sample split of the sub-data-set of Table 3 is shown in Table 4.

(6) Estimate the nonadditive set functions from the training set as follows:

(a) Compute and set the components of the matrices leading to the nonadditive set functions:

FOR $k = 1, 2, \dots 120$

FOR $j = 1, 2, \dots, 2^n - 1$ (all possible combinations of the $1, 2, \dots, n$ chosen attributes) perform the following steps:

i. Compute $z_{kj} = \min_{i:frc(j/2^i)\in[1/2,1)} f(x_i) - \max_{i:frc(j/2^i)\in[0,1/2)} f(x_i)$ if it is positive or $j = 2^n - 1$, and $z_j = 0$ otherwise. Here frc stands for fractional part.

TABLE 4. Additional file 1: Table S4. Sample split of the sub-data-set of Table 3 in a training set of size 120 and a testing set of size 10 for the given initial state j.

Node	x_1	x_2	x_6	Target	
1	$f_1(x_1)$	$f_1(x_2)$	$f_1(x_6)$	AHD(1,NumberInitialState)	Training set
2	$f_2(x_1)$	$f_2(x_2)$	$f_2(x_6)$	AHD(2,NumberInitialState)	
...	...	...	...	...	
120	$f_{120}(x_1)$	$f_{120}(x_2)$	$f_{120}(x_6)$	AHD(120,NumberInitialState)	
121	$f_{121}(x_1)$	$f_{121}(x_2)$	$f_{121}(x_6)$	AHD(121,NumberInitialState)	Testing set
...	...	...	...	...	
130	$f_{130}(x_1)$	$f_{130}(x_2)$	$f_{130}(x_6)$	AHD(130,NumberInitialState)	

ii. Set $\mu_j = \mu(\bigcup_{i:j_i=1}\{x_i\}) = \mu^+(\bigcup_{i:j_i=1}\{x_i\}) - \mu^-(\bigcup_{i:j_i=1}\{x_i\})$ if j, expressed in terms of binary digits $j_n, j_{n-1}, \ldots, j_1$ for every j ($\mu = \mu^+ - \mu^-$).

iii. Set $(C)\int f_k\, d\mu = \sum_{j=1}^{120} z_{kj}\mu_j$.

END FOR LOOP $j = 1, 2, \ldots 2^n - 1$

END FOR $k = 1, 2, \ldots 120$

(b) Using the matrices $Z = [z_{kj}], M = [\mu_j]$ and $AHDmatrix = [AHD(k, NumberInitialState)]$, solve for M such that $||ZM - AHDmatrix|| = \sum_{k=1}^{120}[(C)\int f_k d\mu - AHD(k, NumberInitialState)]^2$ is minimal. Recall that the actual details of implementation and effectiveness of the algebraic method can be found in Reference [17] of the main text.

(7) Use the estimated nonadditive set functions μ to generate the estimated target values $\hat{A}HD(121, NumberInitialState), \ldots, \hat{A}HD(130, NumberInitialState)$ via the equation $ZM = Y$ generated over the testing set.

END FOR LOOP NumberInitialState $= 1, 2, \ldots 100$

Validation using the average error.

(8) Denote by $AHD_{NumberInitialState,j,l}$ the target value corresponding to a given initial state, the jth combination of attributes, and the lth line of the testing set. Compute the average error over all initial states and the entire testing set of size L, for each of the $j = 1, 2, \ldots, 91$ combinations of attributes corresponding to $n = 3, 4, 5$

(could be used only with one single choice of n).

$$E_j = \frac{1}{100} \sum_{NumberInitialState=1}^{100} Error_{NumberInitialState,j}$$

where

$$Error_{NumberInitialState,j} = \frac{1}{L} \sum_{l=1}^{L} |AHD_{NumberInitialState,j,l} - \hat{A}HD_{NumberInitialState,j,l}|$$

for $NumberInitialState = 1, 2, \ldots, 100, j = 1, 2, \ldots, 91, l = 1, 2, \ldots, L$.

(9) Plot these errors as in Figure 4 of the main text to identify their magnitude. The smaller the errors the better the estimation, and that validates the method as in Section 4.1 of the main text.

Best combination of attributes.

(10) Find the best combination of attributes as follows:

i. Compute the consistency in target values
$CT_j = std\left[Error_{NumberInitialState,j}\right]_{NumberInitialState}, j = 1, 2, \ldots 91$
where $std[\cdot]_{NumberInitialState}$ stands for standard deviation over the 100 different initial states.

ii. Compute the consistency in nonadditive set functions
$CM_j = \frac{1}{2^n-1} \sum_{i_1,i_2,\ldots,i_m} S_{i_1 i_2 \ldots i_m}$, with
$S_{i_1 i_2 \ldots i_m} = std\left[\mu(\{x_{i_1}, x_{i_2}, \ldots, x_{i_m}\})\right]_i, m = 1, 2, \ldots NumberAttributes$ where $1 <= i_1 <= i_2 <= \cdots <= i_m <= NumberAttributes$ identify the collection of attributes for which we compute the nonadditive set function, and $NumberAttributes$ is the number of attributes in the jth combination of attributes. For a given $NumberAttributes$ there are $2^{NumberAttributes} - 1$ such collections of indices.

iii. Find the index $J \in \{1, 2, \ldots, 91\}$ of the *best combination of attributes* given by $J = \arg\min_j \left[\sqrt{E_j^2 + CT_j^2 + CM_j^2}\right]$.

End of algorithm
